# Analysis of current situation and influencing factors of marital adjustment in patients with Crohn’s disease and their spouses

**DOI:** 10.1097/MD.0000000000037527

**Published:** 2024-03-15

**Authors:** Ting Pan, Danlei Chen, Zhihui Yu, Qing Liu, Yan Chen, Ailing Zhang, Fang Kong

**Affiliations:** a Nanjing University of Chinese Medicine, Nanjing, China; b Department of Nursing, Nanjing Hospital Affiliated to Nanjing University of Chinese Medicine (Nanjing Second Hospital), Nanjing, China; c Nanjing Hospital Affiliated to Nanjing Medical University (Nanjing First Hospital), Nanjing, China; d Digestive Disease Treatment Center, Nanjing Hospital Affiliated to Nanjing University of Chinese Medicine (Nanjing Second Hospital), Nanjing, China.

**Keywords:** Crohn’s disease, influencing factors, marital adjustment, nursing, spouse

## Abstract

The purpose of this study was to investigate the marital adjustment of patients with Crohn’s disease and their spouses, and to analyze its influencing factors. It lays the investigation foundation for the follow-up binary study of Crohn’s disease patients and their spouses. Using convenience sampling, 177 pairs of patients and their spouses from a tertiary hospital in Nanjing, China were selected. With face-to-face electronic questionnaires to survey the patient and spouse, the contents include the Lock-Wollance Marriage Adjustment Test, Subjective Well-Being Scale for Chinese Citizens, Couple Support Questionnaire, and Distress Self-Disclosure Scale. The marital adjustment score of patients was (99.03 ± 24.25), and the marital adjustment score of spouses was (99.61 ± 25.39). The proportions of patients with marital disorders and their spouses with marital disorders were 52.5% and 46.9%, respectively. Multiple linear regression showed that the spouse’s age, family monthly income, time of diagnosis of Crohn’s, distress self-disclosure, marital support, and subjective well-being were important factors influencing the marital adjustment of patients. Self-disclosure of spousal distress, marital support, age, and subjective well-being were important factors that influenced spouses’ marital adjustment. Most couples with Crohn’s disease have marital disorders, and their marital adjustment affects each other. However, the assessment results of one partner should not be limited to replacing those of the couple. In clinical practice, patient age, monthly family income, self-disclosure of distress, marital support, and subjective well-being should be considered. Spouses should be encouraged to participate in patient care and patient–spouse interventions should be implemented as a whole to improve marital stability.

## 1. Introduction

Crohn’s disease (CD) is a subtype of inflammatory bowel disease, which has a prolonged course and a tendency of lifelong recurrence. It is a disabling disease^[1]^ that seriously affects the daily work and life of patients. CD has a high incidence in European and North American countries, and although the incidence in Asia is lower than that in Western countries,^[2]^ its incidence and prevalence have also shown a steady increase in recent years, and CD has become a global disease. Studies have shown that China is currently one of the countries with a high incidence of CD in Asia,^[3]^ which has been increasing in recent years, and the number of inflammatory bowel disease (IBD) patients in China is expected to exceed 1.5 million in 2025.^[1]^ The diagnosis, treatment, and nursing of the disease should be widely concerned. Limited by medical conditions, CD cannot be cured at present, and the patient’s body and mind are greatly harmed. With the development of medical technology, active treatment and nursing can effectively improve the prognosis of patients, control symptoms, and make patients gradually realize that CD is a chronic disease that can be treated and controlled. This change of understanding not only has a positive impact on the care of CD patients but also allows the scope of care to be extended from treatment and rehabilitation to patient life and psychological state.

However, CD is called “green cancer,” which can persist for a lifetime. CD can be challenging both for the person accepting the reality of the disease and for those living with it, especially for married patients, and it can also bring great stress to spouses and families. As the main group in the family, spouses have great potential to influence the developmental outcome of patients. Studies have shown that patients with IBD^[4]^ have significantly higher rates of separation and divorce than the general population.85% of the patients require nutritional therapy,^[5]^ and the economic burden is serious.^[6]^ Some patients have stoma and nasogastric feeding tubes, which lead to body image disorders, and 50% of patients experience negative emotions, such as anxiety and depression.^[7]^ Married couples of childbearing age are more likely to have psychological problems,^[8]^ and both parties will face more marriage problems, which are important factors that threaten the marriage life of couples with CD and have adverse effects on their marriage adjustment.

How to improve the marital situation of patients, coordinate the relationship between patients and their spouses, and improve the family happiness of patients has become a public health problem that we medical staff need to face together. Marital adjustment refers to the mutual adaptation of husbands and wives to achieve harmony in a certain period,^[9]^ reflecting adjustment and coping ability when the marriage relationship changes. Those with higher marital adjustment ability^[10]^ can better cope with changes in the relationship between husbands and wives when diseases or disease changes occur, to increase the stability of marriage. Relevant studies have shown^[11]^ that good marital adjustment can not only meet the needs of patients and their spouses for love and belonging, bring support and comfort to both parties, relieve the negative emotions of both spouses, but also promote the adjustment and adaptation of both spouses to stressful events, improve the health outcome of patients, and have positive significance for the outcome of the disease. Therefore, it‘s important and necessary to strengthen the research on the marital adjustment of CD patients and their spouses, because it is beneficial to help maintain the marital relationship of patients.

Most of the previous studies focused on CD patients themselves, such as enteral nutrition,^[12]^ exercise,^[13]^ and sleep^[14]^ in CD patients. However, there is no research on marriage adjustment from the dyadic perspective of CD patients and their spouses, and joint research on both husband and wife is necessary. Therefore, this study investigates the marriage adjustment of CD patients and their spouses from a dyadic perspective, investigates the current situation of marriage adjustment of CD patients and their spouses, and analyzes its influencing factors, to provide a theoretical basis for clinical coordination of the marriage relationship between CD patients and their spouses.

## 2. Objects and methods

### 2.1. Objects

Convenience sampling was used to select patients with CD and their spouses from December 2022 to June 2023 in a Grade A hospital in Nanjing as the investigation object. In China, due to technical limitations, not all hospitals have the ability to accept Crohn’s disease, and patients need to go to a specific hospital for medical treatment. This tertiary class A hospital in Nanjing participated in the study, and all participants were CD patients admitted to the inflammatory bowel disease specialty of the Department of Gastroenterology of this hospital. This hospital is famous for having authoritative experts and cutting-edge treatment techniques in the field of CD, and in this hospital, this department treats more than 30 CD patients every day. The patients came from different regions, which had certain regional representation and universality.

Power and sample size estimations were performed using the G.power (3.1 version) software. The sample size was 126 (one group included one couple). The final sample size of the 139 groups was determined to allow for a 10% loss to follow-up. Inclusion criteria for patients were as follows: ① in accordance with the diagnostic criteria for CD formulated by the IBD Group of the Chinese Society of Gastroenterology in 2018^[15]^ (Patients with hospital-confirmed diagnosis of CD, and patients with gastrointestinal endoscopy and immunology confirmation); ② married, having a spouse; ③ informed consent to participate in the study; ④ primary school education or above; ⑤ ability to use electronic information products (such as smartphones) independently and understand electronic questionnaires. The exclusion criteria were: ① language deficit and cognitive impairment; ② serious illness such as heart, brain, kidney, or other malignant tumors, inability to communicate, history of mental illness, or current use of antipsychotic medications. The inclusion criteria were as spouses: ① living with the patient and knowing the patient’s condition; ② informed consent to participate in the study; ③ primary school education or above; ④ the ability to use electronic information devices (such as smartphones) independently and read and understand electronic questionnaires. Exclusion criteria for spouses were: ① language deficit and cognitive impairment; ② serious illness such as heart, brain, kidney, or other malignant tumors, inability to communicate, history of mental illness, or current use of antipsychotic medications. Patients and their spouses were included in the study if they met the inclusion criteria.

### 2.2. Survey tools

A general information questionnaire was prepared by the researchers. The main information included age, ethnicity, sex, duration of marriage, residence, education, occupation, work (or study) status, monthly family income, number of children, payment of medical expenses, and disease (disease status, course of the disease, operation, whether with a stoma, nasogastric tube, or double abdominal tube) of the patients and their spouses.

The research tools used in this study have been translated into Chinese and evaluated by the index of item objective congruence of 3 experts. The results showed that each item was >0.5. The test–retest reliability of the Chinese version of the scale was tested.

The Locke-Wollance Marital Adjustment Test^[16]^ was translated into Chinese in 1999 to evaluate the degree of intimacy and marital happiness among married couples. The scale had 15 items. The total score of the questionnaire was the sum of the scores for each item, ranging from 2 to 158. The higher the score, the higher the intimacy and happiness of the marriage. A score of <100 points was defined as a marital disorder and ≥100 points as a good marital adjustment. The internal consistency coefficient of the scale used in this study is 0.76.

The Subjective Well-being Scale for Chinese Citizens was revised into the Chinese version by Chinese scholar Xing Zhanjun^[17]^ in 2002. There were 20 items across 10 dimensions: content and abundance, mental health, social confidence, growth and progress, goal value, self-acceptance, physical health, mental balance, interpersonal adaptation, and family atmosphere. The internal consistency coefficient of the scale used in this study is 0.86.

Shuyue compiled the Couple Support Questionnaire by Shuyue^[18]^ in 2008 to measure spousal support obtained in marriage relationships. The scale is divided into 2 dimensions, subjective and objective, with a total of 18 items. Higher scores indicate better marital support. The internal consistency coefficient of the scale used in this study was 0.91.

The Chinese version of the Distress Disclosure Index^[19]^ was revised in 2009. Twelve items were included in the study. The Distress Disclosure Index was scored using a 5-point Likert scale ranging from 12 to 60, with higher scores indicating higher levels of individual self-disclosure. The internal consistency coefficient of the scale used in this study is 0.78.

### 2.3. Data collection

The survey was conducted by face-to-face electronic questionnaire distribution. Before the initiation of the study, researchers distributed printed informed consent forms to patients and their spouses, explained the purpose, significance, and privacy protection of the study to patients, and obtained patients’ consent to sign the informed consent form in person. A researcher distributed the two-dimensional code containing the questionnaire items, and participants completed the questionnaire by scanning the code face-to-face using their smartphones. The questionnaire was compiled using the Sojump electronic software and distributed by link or two-dimensional code. During the filling out of the questionnaire, patients and their spouses completed the questionnaire independently and truthfully and were not allowed to exchange information with each other during the completion period. The researcher was on hand to help solve the problem. Participants were asked to complete the full questionnaire and submit it on the spot; incomplete questionnaires could not be submitted online, in which way we could collect patient responses to all questions. During the study, researchers assisted those who could not perform this task independently. The questionnaire was collected by another researcher after returning to the platform. All the same answers or questionable items were collected by patients on the spot. If the patients did not explain, the questionnaire would be removed. A total of 190 groups of questionnaires were distributed and 177 valid questionnaires were returned, with an effective recovery rate of 93.1%.

### 2.4. Data analysis

Excel for Windows (22.0 version) was used to organize the data. The data were analyzed using IBM SPSS for Windows, version 26.0.The measurement data were described by the average ± standard, and the counting data were described by frequency and percentage. Differences in marital adjustment scores between the patients and their spouses were analyzed using a single-sample *t*-test. Count data were expressed as n (%) and compared by *t*-test. In the univariate analysis of marital adjustment of patients and their spouses, an independent sample *t*-test was used for the categories of ≤2 groups, and ANOVA analysis was used for the categories of ≥3 groups. Pearson correlation analysis was used for bivariate correlation analyses. The multiple linear regression model is used to analyze the linear relationship between the explained variable and several other explanatory variables. Multiple linear regression analysis was used for multivariate analysis of marital adjustment between patients and their spouses. Power and sample size estimations were performed using the G.power (3.1 version) software. A medium effect size *f*^2^ = 0.3, *α* = 0.05, and power 1-β = 0.8 were used. *P* < .05 was considered statistically significant.

## 3. Result

### 3.1. General information

The average age of the 177 CD patients was (40.64 ± 9.48) years old. All patients were of yellow race, and 97.8% of the patients were of Han nationality. The Marital Adjustment Test (MAT) scores of patients with a CD of different ages, residence, education, occupation, work (study) status, family monthly income, payment of medical expenses, and time of disease diagnosis were compared, and the differences were statistically significant (*P* < .05), as shown in Table 1.

**Table 1:** Single factor analysis of general data of Crohn’s disease patients and marital adjustment (n = 177).

Characteristic	Examples	MAT score	*t/F*	*P*
Gender			0.71	.401
Male	104 (58.8%)	99.11 ± 23.70		
Female	73 (41.2%)	98.92 ± 25.18		
Age			2.527	.043
20–29	19 (10.7%)	93.26 ± 28.60		
30–39	69 (39.0%)	99.22 ± 23.54		
40–49	58 (32.8%)	100.19 ± 22.18		
50–59	25 (14.1%)	104.20 ± 23.73		
≥60	6 (3.4%)	72.67 ± 27.67		
Residence			6.425	.002
Urban areas	91 (51.4%)	105.13 ± 24.37		
Towns	41 (23.2%)	93.90 ± 23.17		
Rural areas	45 (25.4%)	91.36 ± 22.07		
Duration of marriage			0.374	.772
1–5 years	27 (15.3%)	97.78 ± 27.43		
6–10 years	43 (24.3%)	97.33 ± 22.39		
11–15 years	25 (14.1%)	96.56 ± 25.10		
Over 16 years	82 (46.3%)	101.09 ± 24.11		
Education			6.272	.000
Junior high school and below	71 (40.1%)	90.01 ± 22.01		
High school or secondary school	34 (19.2%)	100.74 ± 20.45		
College or above	72 (40.7%)	107.11 ± 25.23		
Occupations			7.462	.000
Employees of enterprises and institutions	50 (28.2%)	111.94 ± 22.83		
Worker	17 (9.6%)	98.76 ± 22.77		
Farmer	26 (14.7%)	83.54 ± 23.73		
Retirement	33 (18.6%)	97.15 ± 20.82		
Self-employed or freelance	51 (28.8%)	95.57 ± 21.14		
Work (Study) status			11.348	.000
Keeping working (study)	39 (22.0%)	111.33 ± 26.50		
Alternating work (study) and sick leave	35 (19.8%)	105.20 ± 21.29		
Long-term sick leave or unemployment	103 (58.2%)	92.27 ± 22.01		
Monthly family income (RMB)			23.608	.000
≤3000	53 (29.9%)	83.17 ± 21.75		
3001–6000	55 (31.1%)	97.47 ± 18.35		
6001–10,000	33 (18.6%)	104.33 ± 20.52		
≥10,001	36 (20.3%)	119.89 ± 22.15		
Number of children			2.243	.085
No children	16 (9.0%)	108.06 ± 26.54		
The only child	120 (67.8%)	100.28 ± 23.10		
Two children	33 (18.6%)	93.12 ± 26.02		
Three children or above	8 (4.5%)	86.63 ± 23.48		
Primary caregiver			1.047	.353
Spouse	132 (74.5%)	100.54 ± 24.64		
Children	6 (3.4%)	92.00 ± 11.05		
Oneself	39 (22.0%)	95.00 ± 24.13		
Payment of medical expenses			3.949	.048
Self-financing	22 (12.4%)	92.68 ± 17.01		
Medical insurance	155 (87.6%)	99.79 ± 24.91		
Time of diagnosis			3.092	.048
<5 years	64 (36.2%)	104.70 ± 25.17		
5–10 years	57 (32.2%)	97.49 ± 20.65		
>10 years	52 (31.6%)	94.11 ± 25.64		
Disease states			0.136	.713
Remission	129 (72.9%)	101.01 ± 23.97		
Episodes	48 (27.1%)	93.71 ± 24.43		
Surgical conditions			0.916	.34
Once had surgery	48 (27.1%)	100.50 ± 22.74		
Never had surgery	129 (72.9%)	98.48 ± 24.85		
The presence or absence of a stoma			1.36	.245
Yes	26 (14.7%)	94.65 ± 19.99		
No	151 (85.3%)	99.78 ± 24.89		
The presence or absence of a nasogastric tube			1.18	.279
Yes	77 (43.5%)	92.71 ± 25.96		
No	100 (56.5%)	103.89 ± 21.75		
Whether there was a double cannula in the abdominal cavity			1.778	.184
Yes	4 (2.3%)	90.50 ± 11.82		
No	173 (97.7%)	99.23 ± 24.44		

MAT = marital adjustment test.

The average age of the spouses of 177 CD patients was (40.75 ± 9.74) years old. All the spouses were of yellow race and all were of Han nationality. There were statistically significant differences (*P* < .05) in MAT scores of spouses of CD patients with different ages, education levels, occupations, and work (learning) states. Further details are provided in Table 2.

**Table 2:** Single factor analysis of general data of Crohn’s disease spouses and marital adjustment (n = 177).

Characteristic	Examples	MAT score	*t/F*	*P*
Gender				
Male	76 (42.9%)	101.46 ± 23.71	0.840	.402
Female	101 (57.1%)	98.22 ± 26.61		
Age			4.188	.003
20–29	17 (9.4%)	83.82 ± 34.10		
30–39	71 (40.1%)	97.15 ± 23.57		
40–49	58 (32.8%)	103.79 ± 23.51		
50–59	24 (13.6%)	100.67 ± 22.13		
≥60	7 (4.0%)	124.57 ± 22.73		
Education			6.211	.002
Junior high school and below	64 (36.2%)	91.48 ± 27.70		
High school or secondary school	38 (21.5%)	108.26 ± 24.43		
College or above	75 (42.4%)	102.16 ± 21.88		
Occupations			2.922	.023
Employees of enterprises and institutions	64 (36.2%)	107.63 ± 21.34		
Worker	35 (19.8%)	92.34 ± 24.68		
Farmer	16 (9.0%)	92.37 ± 29.79		
Retirement	15 (8.5%)	99.47 ± 31.82		
Self-employed or freelance	47 (26.6%)	96.62 ± 25.26		
Work (Study) status			3.364	.037
Keeping working (study)	123 (69.5%)	102.83 ± 24.84		
Alternating work (study) and sick leave	23 (13.0%)	91.22 ± 23.57		
Long-term sick leave or unemployment	31 (17.5%)	93.06 ± 26.91		

MAT = marital adjustment test.

### 3.2. Current status of MAT, subjective well-being, distress self-disclosure, and couple support of CD patients and their spouses

Sexual relationships were assessed on a score of 8. See Table 3 for details.

**Table 3:** Crohn’s disease couples the scale scores.

Projects	Patients (n = 177)	Spouses (n = 177)
Marital adjustment score	99.03 ± 24.25	99.61 ± 25.39
Subjective well-being score	81.20 ± 14.75	83.97 ± 13.95
Distress self-disclosure score	39.81 ± 7.83	40.93 ± 7.39
Couple support score	103.62 ± 2 0.02	91.67 ± 30.18
>100 points	84 (47.5%)	94 (53.1%)
≤100 points	93 (52.5%)	83 (46.9%)

### 3.3. Correlation analysis of MAT with distress self-disclosure, couple support, and subjective well-being in CD patients and their spouses

Subjective well-being, distress self-disclosure, and couple support of patients with CD and their spouses were positively correlated with MAT (*P* < .05). Although there was no correlation between the dimensions of social confidence and the growth progress of patients’ subjective well-being and MAT (*P* > .05), the other items were significantly correlated with MAT (*P* < .05). The dimensions of social confidence, growth progress, and self-acceptance in the subjective well-being of spouses of patients with CD did not correlate with their spouses’ MAT (*P* > .05). In contrast, other parameters were significantly correlated with MAT (*P *< .05), as shown in Table 4.

**Table 4:** Correlation between each scale and marriage adjustment test of Crohn’s disease patients and spouses (*r*).

Projects	Patients (n = 177)	Spouses (n = 177)
Distress self-disclosure total score	0.26**	0.59**
Couples support total score	0.35**	0.80**
Subjective support	0.38**	0.37**
Objective support	0.28**	0.36**
Subjective well-being total score	0.33**	0.28**
Abundant content	0.27**	0.22**
Mental health	0.27**	0.24**
Social confidence	0.10	0.12
Growth progress	0.13	0.67
Target value	0.24**	0.21**
self-acceptance	0.16*	0.11
Good health	0.22**	0.25**
Mental balance	0.31*	0.20**
Interpersonal adjustment	0.25**	0.20**
Family atmosphere	0.27**	0.26**

* At level .05 (two-tailed), the correlation was significant.

** At level .01 (two-tailed), the correlation was significant.

### 3.4. Multivariate linear regression analysis of influencing factors of MAT in CD patients and spouses

The dependent variable in this study was the total MAT score, and 19 variables with statistical significance in the univariate and correlation analyses were used as independent variables for the multiple linear regression analysis. Continuous variables were assigned Arabic numerals (with size attributes), and disordered categorical variables were treated as dummy variables before the stepwise regression analysis. Table 5 presents the assignments of the independent variables.

**Table 5:** Description of the assignment of argument variables.

Independent variable	Assignment case
Age	20–29 = 1; 30–39 = 2; 40–49 = 3; 50–59 = 4; ≥60 = 5
Residence	Take rural areas as the reference, Urban areas = (0,1); Towns = (0,1);
Education	Junior high school and below = 1, High school or secondary school = 2; College or above = 3
Occupations	Take employees of enterprises and institutions as the reference; Worker = (0,1); Farmer = (0,1); Retirement = (0,1); Self-employed or freelance = (0,1)
Work (Study) status	Take keeping work (study) as a reference; Alternating work (study) and sick leave = (0,1); Long-term sick leave or unemployment = (0,1)
Monthly family income (RMB)	≤3000 = 1; 3001–6000 = 2; 6001–10,000 = 3; ≥10,001 = 4
Payment of medical expenses	Self-financing = 0; Medical insurance = 1
Time of diagnosis	<5 years = 1; 5–10 years = 2; >10 years = 3

The weight of the independent variable was ranked from high to low, according to the absolute value of the standardized regression coefficient. The results of the multiple linear regression analysis of the influencing factors of MAT in patients with CD showed that monthly family income, time of CD diagnosis, distress self-disclosure, age, subjective well-being, and couple support were included in the regression equation, which could explain 42.9% of the total variation (Table 6).

**Table 6:** Multiple regression analysis of influencing factors of marriage adjustment test in Crohn’s disease patients (n = 177).

Independent variable	Regression coefficient	Standard error	Standardized regression coefficient	*t*	*P*
Monthly family	8.332	1.592	0.38	5.235	.000
Time of diagnosis	−4.308	1.886	−0.146	−2.284	.024
Distress self-disclosure	0.517	0.195	0.167	2.648	.009
Age	0.461	0.189	0.180	2.443	.016
Subjective well-being	0.272	0.107	0.167	2.539	.012
Couple support	0.204	0.078	0.169	2.61	.010

*R^2^* = 0.481, adjusted *R^2^* = 0.429, *F* = 9.271, *P < *.001.

The multiple linear regression analysis of the influencing factors of MAT for CD spouses showed that distressed self-disclosure, couple support, age, and subjective well-being entered the regression equation, which could explain 70.4% of the total variation (Table 7).

**Table 7:** Multiple regression analysis of influencing factors of marriage adjustment test in Crohn’s disease spouses (n = 177).

Independent variable	Regression coefficient	Standard error	Standardized regression coefficient	*t*	*P*
Spouses’ distress self-disclosure	0.772	0.166	0.225	4.648	.000
Spouses’ couple support	0.506	0.043	0.601	11.81	.000
Spouses’ age	0.423	0.120	0.162	3.514	.001
Spouses’ subjective well-being	0.164	0.081	0.090	2.021	.045

*R^2^* = 0.723, adjusted *R^2^* = 0.704, *F* = 39.124, *P < *.001.

## 4. Discussion

### 4.1. The marital adjustment status of CD couples

The study of marriage quality began in 1929 with the publication of “Marriage Research” by sociologist Hamilton. The most commonly used concepts in marital quality research are marital satisfaction and marital adjustment. Marital satisfaction refers to the degree of satisfaction that a couple expects each other to achieve. It is often regarded as a subjective concept, that is, married people’s perceptual perception and experience of marriage. Marital adjustment refers to the harmonious adaptation of husband and wife in a certain period, which is one of the important indicators to objectively evaluate the quality of marriage.

This study used the theory of marriage adjustment school to objectively evaluate the marital quality of CD patients and their spouses. The results showed that the total MAT score of CD couples was (99.32 ± 24.79), which was lower than that of ordinary couples,^[20]^ and also lower than that of breast cancer couples.^[21]^ The results indicate that CD patients’ disease will hurt the marital quality of the couple. The CD is known as a “green tumor,” which can occur in all ages, and the condition is repeated. At present, there is no ideal radical treatment. As a stressful event in the family, it not only affects the patient’s physiological changes but also has a huge impact on the psychological status of both spouses and their families, affecting the marital communication of both husband and wife. The results of the study showed that there were more CD patients with marital disorders than spouses. It is speculated that the reason may be related to the majority of patients in the survey. Studies have shown that^[22]^ high marriage quality depends more on the role performance of men, and the instrumental role function of men in marriage plays an important role in marriage. However, patients bear more disease symptom burden than their spouses,^[23]^ and their role function is reduced, which has a direct adverse effect on marital adjustment.

### 4.2. Analysis of influencing factors of marital adjustment in CD patients

The results showed that the family’s monthly income was the most important factor affecting the patients’ marital adjustment. Economic income directly affects the basic quality of life and medical quality of patients.^[24]^ Most of the patients were working with illness and had high work pressure. This is consistent with findings in breast cancer patients.^[25]^ With the progression of the disease, the patient’s treatment cost increases but the workability decreases. When the expenditure exceeds the income, the patient’s sense of self-burden and family guilt increases,^[26]^ which directly affects the communication between husband and wife, and it is easy to ignore the spiritual aspects such as marriage. Age is one of the factors affecting the marital adjustment of patients. The study found that the older the age, the better the marital adjustment ability. With the increase in the patient’s age, the husband and wife have a richer life and social experience. After continuous run-ins, they become more mature and calm in the face of life changes and can gradually adapt to the common pressure.^[27]^ The ability of marital adjustment is higher. This has also been certified in patients with fibromyalgia.^[28]^ The time of CD diagnosis is also one of the factors affecting marital adjustment. The study found that the longer the time of CD diagnosis, the lower the marital adjustment ability. Due to its incurable and chronic nature, CD will increase the economic and psychological burden of patients, resulting in the impairment of the physical and mental health of patients.^[29]^ Patients are prone to excessive criticism and control behaviors, easily have low satisfaction with life, and seriously affect marriage adjustment.

Distress self-disclosure, marital support, and subjective well-being are important factors affecting the marital adjustment of patients. (1) Self-disclosure refers to the process of individuals sincerely sharing their inner feelings and thoughts with close others (spouses and relatives), which is an objective index to evaluate the level of self-disclosure of patients and their spouses. Patients who express their inner feelings more to their spouses can reduce negative emotions,^[30]^ help release stress, form a virtuous circle in the relationship, and have a stronger ability to deal with problems in the face of marital changes. Proper display of the inner world is more helpful in promoting the communication between husband and wife, increasing the understanding of both sides on the emotional and cognitive levels, promoting the maintenance of close relationships, and improving the adjustment of marriage. This is consistent with the findings of cancer patients.^[31]^ (2) Marital support refers to the supportive behavior that enables the other party to get help, and feel loved, affirmed, and valued in the marriage relationship, which is divided into subjective support and objective support. This study has shown that the more support patients receive from their spouses, the higher their marital adjustment ability. This is consistent with the findings of bladder cancer patients.^[32]^ Spouse’s support for patients can promote patients’ expression and dependence on their spouses, increase patients’ positive evaluation of their spouses, reduce patients’ psychological distress, and make it easy to show stronger marital adjustment ability. Studies have also confirmed^[33]^ that marital support has a positive impact on the increase of intimacy. (3) Subjective well-being is an individual’s overall evaluation of life quality based on self-established standards. The study shows that the subjective well-being of patients is positively correlated with the ability of marital adjustment. Patients with high subjective well-being have stronger emotional self-management.^[34]^ They will cultivate their own positive emotions and accept negative physical and mental problems,^[35]^ so they can make more rational decisions in the face of marriage problems. The stable heart of patients is more conducive to calm and calm to deal with problems, and their marital adjustment ability is higher.

This study found that whether the patients carried a stoma or nasogastric tube did not affect the marital adjustment of the patients. This is inconsistent with previous studies. Patients with a stoma or nasogastric tube are prone to conflict between spouses,^[36]^ which has adverse effects on marital adjustment. This may be related to the differences in study subjects and methods. The number of patients with stoma (26, 14.7%) included in this study was small.

### 4.3. Analysis of influencing factors of marital adjustment of CD spouses

Spouse self-disclosure is the most important influencing factor of marital adjustment. This study found that spouse distress self-disclosure scores were higher than patient distress self-disclosure scores. It may be related to the fact that the spouse not only bears more economic and care burdens in the family system than the patient^[32]^ but also faces the uncertainty of the future caused by the patient’s disease progression, so the spouse needs to talk more than the patient. Clinicians and nurses have fewer opportunities to obtain self-disclosure from patients during their medical visits so they cannot understand patients’ ability to adjust to marriage and family. This suggests^[37]^ that medical workers need to increase empathy and understanding, establish effective self-disclosure conditions, do good listening and guidance, and improve and understand the patients’ marital adjustment ability and mental state from the perspective of their spouses.

Regression analysis showed that age, marital support, and subjective well-being were the important factors affecting marital adjustment. (1) The older the spouse is, the better the marital adjustment ability is. With age and disease progression, the tolerance and understanding of the spouse facing life and the patient will increase,^[38]^ which has a positive impact on the improvement of the marital relationship. (2) There was a positive correlation between spousal support and spousal adjustment, and the spousal support score was lower than that of patients. The possible reason is that the patients who are troubled by the disease pay more attention to the treatment and rehabilitation of the disease, their physical and mental workability is reduced, and they have no time to take into account the companionship and support of their spouses.^[39]^ Despite this, the spouse’s feeling of support from the patient’s close relationship can improve the spouse’s confidence in facing difficulties and stress,^[40]^ and make them more courageous to face the problems brought by stressful events in the marriage relationship. (3) The higher the SWB score, the higher the spouse’s MAT score. The improvement of subjective well-being has a positive significance for the spouse’s mental adjustment.^[41]^ It is more conducive to the stability of marital relationships by increasing cooperation with patients when coping with disease and changes in marital relationships and maintaining an optimistic attitude to face up to treatment and care.

The study also found that there was a correlation between spouses and patients in pain self-disclosure, marital support, and subjective well-being. This also reminds us that in the process of patient care, the staff must treat CD patients and their spouses as a whole, and neither husband nor wife can be isolated. To pay attention to the psychological changes and marriage assessment of CD couples, and to provide a reliable theoretical basis for the formulation of dyadic intervention plans for couples with marriage disorders.

### 4.4. Limitations

First, the major limitation is the cross-sectional design does not allow making causal inferences. Second, all outcomes were self-reported by study participants. Third, research suggests that low response time of the electronic questionnaire there may be a bigger quality problem. Regarding the average questionnaire response time used in this study and the electronic questionnaire response suggestions,^[42]^ we gave a 5-minute questionnaire exclusion standard, but this standard may lead to the exclusion of some valid questionnaires. Fourth, the small number of subjects included in this study, the single-center study, and the limited range of subject options may have biased the results. Finally, the factors affecting the marital adjustment of couples are diverse and complex. To better understand the marital status of CD couples, it is suggested that more relevant studies of different races and different countries should be carried out in the future, and multi-center studies with large samples should be carried out to better understand the ideas of the target population.

## 5. Conclusions

In conclusion, this study highlights the current status and influencing factors of marital adjustment among CD patients and their spouses. The MAT of CD patients is affected by factors such as age, family monthly income, couple support, distress self-disclosure, subjective well-being, and the time of CD diagnosis. Spouse MAT is affected by age, distress self-disclosure, marital support, and subjective well-being. Health workers should pay attention to daily health assessments and health education. The social support department can provide more social practice activities and psychological support, improve the psychological adjustment ability of patients, and provide more marriage guidance for couples with marriage disorders. As the decision-makers and regulators of the marriage system, the civil affairs department and marriage support agencies need to carry out necessary training and cognitive education on social support for patients and their families, to improve their marriage adjustment. Medical institutions should provide more online and offline training to improve the self-care level of patients, and to improve the marital adjustment of couples.

## Acknowledgments

The authors thank all participants who were involved with this study. Additionally, the authors thank the doctors and nurses in the department for their help.

## Author contributions

**Conceptualization:** Ting Pan, Danlei Chen, Zhihui Yu, Qing Liu, Yan Chen, Ailing Zhang, Fang Kong.

**Data curation:** Zhihui Yu, Qing Liu, Ailing Zhang.

**Formal analysis:** Yan Chen, Fang Kong.

**Supervision:** Yan Chen, Fang Kong.

**Writing – original draft:** Ting Pan, Danlei Chen.

**Writing – review & editing:** Ting Pan, Danlei Chen, Zhihui Yu, Qing Liu, Yan Chen, Ailing Zhang, Fang Kong.

## References

[1] Kaplan GG. The global burden of IBD: from 2015 to 2025. Nat Rev Gastroenterol Hepatol. 2015;12:720–7. 26323879 10.1038/nrgastro.2015.150

[2] Park J Cheon JH. Incidence and prevalence of inflammatory bowel disease across Asia. Yonsei Med J. 2021;62:99–108. 33527789 10.3349/ymj.2021.62.2.99 PMC7859683

[3] Mak WY Zhao M Ng SC. The epidemiology of inflammatory bowel disease: east meets west. J Gastroenterol Hepatol. 2020;35:380–9. 31596960 10.1111/jgh.14872

[4] Ng SC Kaplan GG Tang W. Population density and risk of inflammatory bowel disease: a prospective population-based study in 13 countries or regions in Asia-Pacific. Am J Gastroenterol. 2019;114:107–15. 30177785 10.1038/s41395-018-0233-2

[5] Meima-van Praag EM Buskens CJ Hompes R. Surgical management of Crohn’s disease: a state of the art review. Int J Colorectal Dis. 2021;36:1133–45. 33528750 10.1007/s00384-021-03857-2 PMC8119249

[6] Pakdin M Zarei L Bagheri Lankarani K. The cost of illness analysis of inflammatory bowel disease. BMC Gastroenterol. 2023;23:21. 36658489 10.1186/s12876-023-02648-z PMC9854042

[7] Bisgaard TH Allin KH Keefer L. Depression and anxiety in inflammatory bowel disease: epidemiology, mechanisms and treatment. Nat Rev Gastroenterol Hepatol. 2022;19:717–26. 35732730 10.1038/s41575-022-00634-6

[8] Li G Ren J Wang G. Impact of Crohn’s disease on marital quality of life: a preliminary cross-sectional study. J Crohns Colitis. 2015;9:873–80. 26142464 10.1093/ecco-jcc/jjv121

[9] Bernstein CN Kraut A Blanchard JF. The relationship between inflammatory bowel disease and socioeconomic variables. Am J Gastroenterol. 2001;96:2117–25. 11467642 10.1111/j.1572-0241.2001.03946.x

[10] Jedel S Hood MM Keshavarzian A. Getting personal: a review of sexual functioning, body image, and their impact on quality of life in patients with inflammatory bowel disease. Inflamm Bowel Dis. 2015;21:923–38. 25789923 10.1097/MIB.0000000000000257 PMC4369789

[11] Brandão T Pedro J Nunes N. Marital adjustment in the context of female breast cancer: a systematic review. Psychooncology. 2017;26:2019–29. 28342270 10.1002/pon.4432

[12] Anglim J Horwood S Smillie LD. Predicting psychological and subjective well-being from personality: a meta-analysis. Psychol Bull. 2020;146:279–323. 31944795 10.1037/bul0000226

[13] Ashton JJ Gavin J Beattie RM. Exclusive enteral nutrition in Crohn’s disease: Evidence and practicalities. Clin Nutr. 2019;38:80–9. 29398336 10.1016/j.clnu.2018.01.020

[14] Papadimitriou K. Effect of resistance exercise training on Crohn’s disease patients. Intest Res. 2021;19:275–81. 33207853 10.5217/ir.2020.00043 PMC8322027

[15] Sofia MA Lipowska AM Zmeter N. Poor sleep quality in Crohn’s disease is associated with disease activity and risk for hospitalization or surgery. Inflamm Bowel Dis. 2020;26:1251–9. 31820780 10.1093/ibd/izz258 PMC7365809

[16] Inflammatory Bowel Disease Group, Chinese Society of Gastroenterology, Chinese Medical Association. Evidence-based consensus on opportunistic infections in inflammatory bowel disease (republication). Intest Res. 2018;16:178–93. 29743831 10.5217/ir.2018.16.2.178 PMC5934591

[17] Freeston MH Pléchaty M. Reconsideration of the Locke-Wallace marital adjustment test: is it still relevant for the 1990s? Psychol Rep. 1997;81:419–34. 9354091 10.2466/pr0.1997.81.2.419

[18] Kokoszka A Pacura A Kostecka B. Body self-esteem is related to subjective well-being, severity of depressive symptoms, BMI, glycated hemoglobin levels, and diabetes-related distress in type 2 diabetes. PLoS One. 2022;17:e0263766. 35167598 10.1371/journal.pone.0263766 PMC8846537

[19] Hua Z Mei L Ying-Feng D. Relationship between the fear of cancer recurrence and quality of life among patients with gynecology cancer: mediating effect of spouse support. Chin J Gen Pract. 2019.

[20] Gao RC Wu L Shi PL. The impact of distress disclosure and anxiety on the association between social support and quality of life among Chinese women with systemic lupus erythematosus. Front Psychiatry. 2022;13:893235. 35990077 10.3389/fpsyt.2022.893235 PMC9385970

[21] Yang Y Luo B Ren J. Marital adjustment and depressive symptoms among Chinese perinatal women: a prospective, longitudinal cross-lagged study. BMJ Open. 2023;13:e070234. 10.1136/bmjopen-2022-070234 PMC10619017 37899151

[22] Suo R Ye F Xie M. The relationship of marital adjustment and posttraumatic growth in female breast cancer patients and their husbands. Psychol Health Med. 2023;28:401–7. 35440230 10.1080/13548506.2022.2067339

[23] Ma SS Zhang JT Song KR. Connectome-based prediction of marital quality in husbands’ processing of spousal interactions. Soc Cogn Affect Neurosci. 2022;17:1055–67. 35560211 10.1093/scan/nsac034 PMC9714425

[24] Matthias AT Fernando GVMC Somathilake BGGK. Predictors and patterns of polypharmacy in chronic diseases in a middle-income country. Int J Physiol Pathophysiol Pharmacol. 2021;13:158–65. 35103098 PMC8784655

[25] Namli Z Tamam L Demirkol ME. The relationship among dyadic adjustment and disease burden in patients with bipolar disorder and their spouses. Behav Sci (Basel). 2023;13:91. 36829320 10.3390/bs13020091 PMC9952473

[26] Li M Chan CWH Chow KM. A systematic review and meta-analysis of couple-based intervention on sexuality and the quality of life of cancer patients and their partners. Support Care Cancer. 2020;28:1607–30. 31872299 10.1007/s00520-019-05215-z

[27] Meier A Allen G. Intimate relationship development during the transition to adulthood: differences by social class. New Dir Child Adolesc Dev. 2008:25–39. 18330913 10.1002/cd.207 PMC3703509

[28] Ruiz-Marin CM Molina-Barea R Slim M. Marital adjustment in patients with cancer: association with psychological distress, quality of life, and sleep problems. Int J Environ Res Public Health. 2021;18:7089. 34281026 10.3390/ijerph18137089 PMC8297374

[29] Calandre EP Ordoñez-Carrasco JL Rico-Villademoros F. Marital adjustment in patients with fibromyalgia: its association with suicidal ideation and related factors. A cross-sectional study. Clin Exp Rheumatol. 2021;39 Suppl 130:89–94. 33886451 10.55563/clinexprheumatol/pufzd6

[30] Lee J Gillath O Miller A. Effects of self- and partner’s online disclosure on relationship intimacy and satisfaction. PLoS One. 2019;14:e0212186. 30830918 10.1371/journal.pone.0212186 PMC6398828

[31] Graff LA Fowler S Jones JL. Crohn’s and Colitis Canada’s 2021 impact of covid-19 and inflammatory bowel disease in canada: mental health and quality of life. J Can Assoc Gastroenterol. 2021;4(Suppl 2):S46–53. 34755039 10.1093/jcag/gwab031 PMC8570421

[32] Du X Wang D Du H. The correlation between intimate relationship, self-disclosure, and adaptability among colorectal cancer enterostomy patients. Medicine (Baltimore). 2021;100:e25904. 34106651 10.1097/MD.0000000000025904 PMC8133169

[33] Xiong L Zhou C Yan L. The impact of avoidant attachment on marital satisfaction of Chinese married people: multiple mediating effect of spousal support and coping tendency. Acta Psychol (Amst). 2022;228:103640. 35667243 10.1016/j.actpsy.2022.103640

[34] Cao X Ge R Li X. Factors influencing self-management among patients diagnosed with anxiety disorders in China: a cross-sectional study. J Psychiatr Ment Health Nurs. 2023;30:234–44. 35815835 10.1111/jpm.12857

[35] Falconier MK Kuhn R. Dyadic coping in couples: a conceptual integration and a review of the empirical literature. Front Psychol. 2019;10:571. 30971968 10.3389/fpsyg.2019.00571 PMC6443825

[36] Rachmawati DS Nursalam N Hargono R. Quality of life and subjective well-being modeling of pulmonary tuberculosis patients. J Public Health Res. 2021;10:2180. 33855397 10.4081/jphr.2021.2180 PMC8129758

[37] McDaniel SH Beckman HB Morse DS. Physician self-disclosure in primary care visits: enough about you, what about me? Arch Intern Med. 2007;167:1321–6. 17592107 10.1001/archinte.167.12.1321

[38] Thomeer MB. Multiple chronic conditions, spouse’s depressive symptoms, and gender within marriage. J Health Soc Behav. 2016;57:59–76. 26957134 10.1177/0022146516628179 PMC8258774

[39] Amiot A Chaibi S Bouhnik Y. Prevalence and determinants of fatigue in patients with IBD: a cross-sectional survey from the GETAID. J Crohns Colitis. 2023;17:1418–25. 36988620 10.1093/ecco-jcc/jjad060

[40] Lehane CM Hofsöe SM Wittich W. Mental health and spouse support among older couples living with sensory loss. J Aging Health. 2018;30:1205–23. 28613091 10.1177/0898264317713135

[41] Jebb AT Morrison M Tay L. Subjective well-being around the world: trends and predictors across the life span. Psychol Sci. 2020;31:293–305. 32045327 10.1177/0956797619898826

[42] Zuar LR Chien K Lentine J. Does parenting style affect adolescent IBD transition readiness and self-efficacy scores? Children (Basel). 2021;8:367. 34064470 10.3390/children8050367 PMC8147961

